# Does Emotional Intelligence Mediate the Relation Between Mindfulness and Anxiety and Depression in Adolescents?

**DOI:** 10.3389/fpsyg.2018.02463

**Published:** 2018-12-12

**Authors:** Brigid Foster, Justine Lomas, Luke Downey, Con Stough

**Affiliations:** ^1^Department of Psychological Science, Swinburne University, Melbourne, VIC, Australia; ^2^Emotional Intelligence Research Unit, Swinburne University, Melbourne, VIC, Australia; ^3^Institute for Breathing and Sleep, Austin Hospital, Melbourne, VIC, Australia; ^4^Centre for Human Psychopharmacology, Swinburne University, Melbourne, VIC, Australia

**Keywords:** emotional intelligence, EI, mindfulness, anxiety, depression, adolescents

## Abstract

High anxiety and depression are often observed in the Australian adolescent population, and if left untreated, can have long-term negative consequences impacting educational attainment and a range of important life outcomes. The utilization of mindfulness techniques has been associated with decreased anxiety and depression, but the underlying mechanisms for this is only beginning to be understood. Previous research with adult samples has suggested that the development of emotional intelligence (EI) may be one mechanism by which mindfulness confers its benefits on wellbeing. This study is the first to examine the relation between mindfulness, EI, anxiety, and depression in an adolescent population. It was hypothesized that EI would mediate the relationships between mindfulness and anxiety, as well as mindfulness and depression. The sample consisted of 108 adolescents from a public secondary school, aged between 13 and 15 years (*M*_age_ = 13.68, *SD*_age_ = 0.56, 51 males and 57 females). Participants completed an online self-report questionnaire which measured dispositional mindfulness, EI, anxiety, and depression. The results indicated that one subscale of EI – Emotional Recognition and Expression (ERE) mediated the relation between mindfulness and anxiety, while two subscales of EI – ERE and Emotional Management and Control (EMC) mediated the relation between mindfulness and depression. Future research utilizing a mindfulness intervention should be conducted to examine whether the use of mindfulness increases EI and decreases anxiety and depression in adolescents.

## Introduction

Adolescence is the developmental period between childhood and adulthood and is a time of significant physical, social, and emotional development (Ernst et al., 2006; Garcia, 2010). It is also a time of increased risk-taking and emotional reactivity, combined with comparatively poor decision-making abilities, and impulse control (Steinberg, 2007; Casey et al., 2008). It has been hypothesized that the difference between emotional and cognitive abilities during adolescence explains why this can be a time of increased vulnerability for the onset of affective and anxiety disorders (Steinberg and Morris, 2001; Steinberg, 2005).

Approximately one in seven Australian children and adolescents met the criteria for a diagnosable mental disorder during 2013–2014, with anxiety disorders and major depressive disorder being the most prevalent (Lawrence et al., 2015). Many more adolescents experience sub-clinical symptoms of anxiety and depression (Balazs et al., 2013). These disorders have a significant negative impact on the individual and society, with anxiety and depression both demonstrated to be associated with a range of negative outcomes, including: poorer academic achievement (Mazzone et al., 2007; DeRoma et al., 2009); decreased productivity (Beck et al., 2011; Australian Bureau of Statistics, 2013); decreased subjective wellbeing (Keyes, 2005); increased substance use (Burns and Teesson, 2002); and an increased risk of suicide (Kendall et al., 2004).

Adolescent onset mental disorders can have long-term impacts which extend into adulthood (Goodman et al., 2011). Adolescence is a critical period for identity formation, and a clear and confident self-concept is thought to be an important aspect of psychological well-being (Erikson, 1968). It has been argued that the symptoms of anxiety and depression may interfere with identity formation, thus leading to long-term personality problems (Robins et al., 1996; Akse et al., 2004; van Aken and Semon Dubas, 2007).

Adolescence is also an important time for the completion of education, the acquisition of employment skills, and the development of relationships (American Psychiatric Association., 2002; McNeely and Blanchard, 2010). The disruption to these processes can potentially lead to long-term functional impairment (Costello et al., 1999; Balazs et al., 2013; Peters et al., 2016). Further, those individuals who experience depression or anxiety during adolescence are more likely to have recurrent episodes throughout life (Costello et al., 1999; Kessler et al., 2001; Kendall et al., 2004; Allen et al., 2014).

For these reasons, the identification of effective early interventions to prevent or treat subclinical and diagnosable anxiety and depression in adolescence is of primary importance. Mindfulness has been postulated to be one such intervention and has demonstrated promising results in studies with adults, where higher levels of mindfulness have been demonstrated to be associated with greater psychological wellbeing, and decreased anxiety and depression (Kabat-Zinn, 1994; Shapiro et al., 1998; Brown and Ryan, 2003; Khoury et al., 2013).

Such studies, however, are still rare for the adolescent population and therefore there is a need to evaluate whether a relationship exists between mindfulness and mental health variables. This is one of the aims of the current study where it is hypothesized that greater mindfulness will be associated with decreased depression and anxiety in adolescents, and further, that greater mindfulness will be positively associated with Emotional Intelligence (EI).

### Mindfulness

The Western practice of mindfulness is derived from the practice of meditation which exists in all traditions of Buddhism (Kabat-Zinn, 2003). It can be defined as “paying attention in a particular way: on purpose, in the present moment, and non-judgmentally” (Kabat-Zinn, 1994, p. 4) and has become an increasingly popular practice in the West as it has been demonstrated to be positively associated with psychological wellbeing and self-care, and negatively associated with anxiety and depression in adults (Kabat-Zinn, 1994; Shapiro et al., 1998; Brown and Ryan, 2003; Christopher and Gilbert, 2010; Richards et al., 2010; Khoury et al., 2013).

The practice of mindfulness involves two aspects: attention and attitude. The first aspect involves training the attention toward the present moment by knowing where one’s attention is, prioritizing where one’s attention needs to be, and training one’s attention to stay where it should be (Kabat-Zinn, 2003; Hassed, 2016). The second aspect of mindfulness involves bringing an attitude of openness, curiosity and acceptance to one’s thoughts and observations (Kabat-Zinn, 2003; Hassed, 2016).

Individuals have different levels of dispositional mindfulness (Brown and Ryan, 2003; Baer et al., 2006), but levels of mindfulness can also be increased through practice via mindfulness meditation, which involves gently bringing the attention back to the present moment, while observing but not becoming attached to thoughts (Carmody and Baer, 2008; Hassed, 2016). Participation in a mindfulness-based stress reduction course has been shown to significantly increase levels of mindfulness with effect sizes in the moderate to large range, with the extent of mindfulness practice correlating with the degree of change in mindfulness levels (Carmody and Baer, 2008).

The practice of mindfulness may result in neuroplastic changes to the brain, and these changes are thought to be the basis of the positive effects of mindfulness (Hölzel et al., 2011a,b; Tang et al., 2015). Cross-sectional research has demonstrated that increases in regional gray matter are associated with increased performance abilities (Mechelli et al., 2004), and experienced mindfulness practitioners exhibit greater graey matter concentrations in multiple brain areas compared to those individuals who do not practice mindfulness, including those brain areas involved in attention, learning, memory, and emotional regulation (Hölzel et al., 2011a,b; Tang et al., 2015). Thus, the increase in gray matter concentration in these areas is thought to be one mechanism by which mindfulness confers its benefits (Hölzel et al., 2011b; Paul et al., 2013).

While most of these studies are observational and therefore causation cannot be inferred, a recent randomized control trial found that a 3-day intensive mindfulness meditation training intervention reduced right amygdala resting state functional connectivity indicating that mindfulness meditation promotes neuroplastic changes responsible for a reduction in stress, anxiety and depression (Taren et al., 2015).

Research on mindfulness interventions over the past 20 years has focused on adult clinical populations. More recently there has been increased interest in evaluating effectiveness in mindfulness in children and adolescents (Semple et al., 2005; Hayes and Greco, 2008; Greco et al., 2011; Wootten, 2016).

There is evidence that mindfulness in adolescence differs from that in adults, due to the developing nature of the adolescent brain (Dahl, 2004; Greco et al., 2011). For example, mindfulness measures in adults include items that reflect a *describing* facet which involves the ability to put internal experiences into words, but children and adolescents do not have such well-developed abilities in this area (Baer et al., 2004). Due to these differences, studies that specifically seek to understand mindfulness in adolescents are important, because the developmental period of adolescence strikes a compelling paradox; on the one hand, adolescence is a period of great physical strength and resilience, yet on the other hand, morbidity and mortality rates during adolescence double compared to childhood. The increase in morbidity and mortality rates in adolescence are thought to be due to difficulties in the control of behavior and emotion which lead to higher rates of accidents, suicide, substance use and other risk-taking behavior (Dahl, 2004).

It is therefore important to examine the emotional correlates of mindfulness in adolescence, because mindfulness in adults has been demonstrated to be highly correlated with self-control (Fetterman et al., 2010; Bowlin and Baer, 2012) and emotional regulation (Hill and Updegraff, 2012), two areas where adolescents are under-developed compared to adults, and which result in higher morbidity and mortality (Dahl, 2004).

Because of their age and stage, it is not expected that adolescents will have much formal experience in mindfulness meditation. However, research has demonstrated that individuals differ to the degree to which they are willing or able to attend to what is occurring in the present moment (Brown and Ryan, 2003; Baer et al., 2006) – a fundamental aspect of mindfulness. This is known as dispositional mindfulness. This study uses a self-report measure of dispositional mindfulness which was developed for children and adolescent populations and which assesses such mindfulness skills such as present-centered awareness and a non-judgmental stance toward internal experiences (Greco et al., 2011).

Self-report mindfulness levels in children and adolescents have been found to be associated with greater happiness and satisfaction with life (Brown et al., 2011; Greco et al., 2011), and trait mindfulness has been found to protect against decision-making processes that place adolescents at risk for smoking (Black et al., 2012). Further, higher levels of mindfulness in adolescents have been found to be associated with fewer internalizing and externalizing problems (Greco et al., 2011).

Of most relevance to this manuscript, the few studies on the effects of mindfulness in children and adolescents have found that higher levels of mindfulness are associated with lower depression (Hayes and Greco, 2008; Thompson and Gauntlett-Gilbert, 2008; Broderick and Blewitt, 2012) and anxiety (Semple et al., 2005; Bögels et al., 2008; Hayes and Greco, 2008; Thompson and Gauntlett-Gilbert, 2008).

In adults, there is evidence that mindfulness exerts its beneficial effects on psychological wellbeing by influencing EI. That is, EI has been shown to mediate the relationship between: mindfulness and perceived stress (Charoensukmongkol, 2014; Bao et al., 2015); mindfulness and general self-efficacy (Charoensukmongkol, 2014); mindfulness and life satisfaction and mental distress (Wang and Kong, 2013); and mindfulness and subjective well-being (Schutte and Malouff, 2011). This study will, for the first time, examine the hypothesis that EI mediates the relationship between mindfulness and anxiety and mindfulness and depression in adolescents.

### Emotional Intelligence

Emotional Intelligence (EI) has been defined as “the ability to perceive accurately, appraise and express emotion; the ability to access and/or generate feelings when they facilitate thought; the ability to understand emotion and emotional knowledge; and the ability to regulate emotions to promote emotional and intellectual growth” (Mayer and Salovey, 1997b, p. 10) and despite early controversies as to its validity as a construct (Schulte et al., 2004; Landy, 2005; Locke, 2005) is growing in importance as research demonstrates its association with a number of wellbeing outcomes with medium effect sizes (Schutte et al., 2007). Higher levels of EI are associated with: better physical and mental health (Martins et al., 2010); greater psychological wellbeing (Roberts et al., 2012; Sánchez-Álvarez et al., 2015); academic success (Downey et al., 2008b); less severe depression and social anxiety in clinical samples (Downey et al., 2008a; Nolidin et al., 2013); greater happiness in adolescents (Abdollahi et al., 2015); lower test anxiety among adult students (Ahmadpanah et al., 2016); and lower anxiety and depression in adolescents (Resurrección et al., 2014).

There are two approaches to conceptualizing EI: ability and trait. Mayer et al. (2004) argued that EI is best conceptualized as an ability, in the same way that cognitive intelligence is conceptualized as an ability. In this conceptualization, EI abilities and skills are divided into four branches – the ability to perceive emotions, the ability to use emotions to facilitate thought, the ability to understand emotions and the ability to manage emotion (Mayer et al., 1999; Mayer et al., 2004, 2008b). According to this model, more basic abilities such as perceiving and expressing emotion develop before more complex abilities such as the regulation of emotion (Mayer and Salovey, 1997a). Ability models measure EI by using a set of problem-solving tasks, measured against a criterion of correctness (Mayer et al., 2008b). For example, the Mayer-Salovey-Caruso Emotional Intelligence Test (MSCEIT) assesses the perception of emotion branch by asking participants to identify the emotions in pictures of faces (Mayer et al., 1999).

Trait models view EI as a personality trait (Petrides and Furnham, 2001; Neubauer and Freudenthaler, 2005) and measure EI through self-report questionnaires, although some measures also use rater-versions. Luebbers et al. (2007) developed a self-report and rater adolescent version of the Swinburne University Emotional Intelligence Test (Adolescent SUEIT) which is a self-report measure of EI based on the Mayer and Salovey (1997a) conceptualization of EI as a composite set of abilities. The Adolescent SUEIT broadly supports the four-factor model of EI as proposed by Mayer and Salovey (1997a): understanding and analysing emotions (UE); Emotional recognition and expression (ERE); emotional management and control (EMC); and emotions direct cognition (EDC) (Luebbers et al., 2007).

EI therefore, is not a unitary construct and so it may be most useful and interesting to research how the subscales of EI relate to wellbeing. For example, EMC has been found to be significantly and negatively associated with severity of depression in an adult sample (*r* = -0.56) (Downey et al., 2008a), and in an adolescent sample the four different subscales of EI were differentially associated with anxiety and depression (ERE, *r* = -.21), internalizing (ERE, *r* = -.19, EMC, *r* = -.42) and externalizing (ERE, *r* = -.17, UE, *r* = -.18, EMC, *r* = -.29) behaviors, social problems (UE, *r* = -.17, EMC, *r* = -.32) and coping strategies (ERE, *r* = 0.25, EMC, *r* = 0.32) (Downey et al., 2010). EMC has also been found to be positively associated with greater scholastic success (*r* = 0.25) (Downey et al., 2013) and emotional regulation has been found to be negatively associated with Generalized Anxiety Disorder (*r* = 0.32) (Kerns et al., 2013). EMC has also been found to predict maths (*r*^2^ = 0.06) and science (*r*^2^ = 0.04) results in an adolescent sample, while the UE subscale predicts scores for art (*r*^2^ = 0.12) and geography (*r*^2^ = 0.08) (Downey et al., 2008b). This study uses the Adolescent SUEIT as it has been extensively validated with Australian adolescent populations.

### Mindfulness and EI

Research has demonstrated a positive relationship between mindfulness and some of the subscales of EI, but not others (Brown and Ryan, 2003; Baer et al., 2006; Charoensukmongkol, 2014). There are several proposed explanations for this. Feldman et al. (2006) postulated that mindfulness encourages the present-centered attention to one’s emotions and this coupled with the attitude of non-judgment, leads to a tendency for more mindful individuals to be more self-aware and have a higher clarity to their emotions.

Krasner et al. (2009) hypothesized that because greater mindfulness is associated with better overall attention (Brown et al., 2007), individuals with greater levels of mindfulness are more likely to be more aware of their own emotions. Greater levels of mindfulness are also associated with an enhanced ability to regulate and control emotions (Cahn and Polich, 2006; Broderick and Metz, 2009), increased self-awareness, attention and self-reflection (Jha et al., 2007; Lutz et al., 2008; Krasner et al., 2009) and greater levels of meta-cognitive ability (Zeidan et al., 2010) – the ability to monitor and control thoughts, thought to be the central ability whereby individuals effectively regulate their emotions and a key element of EI (Mayer and Salovey, 1997a). A recent study using the same measure of Adolescent EI as the current study reported significant relationships between adolescent EI scores (specifically Emotional Recognition and Expression (ERE) and Emotional Management and Control (EMC)), dispositional mindfulness and well-being in Australian males (Teal et al., 2018). This study also demonstrated that ERE and EME partially mediated the relationship between dispositional mindfulness and psychological distress which although is different to the measures assessed in the present study provides an excellent platform to hypothesize similar relationships between other measures of mental health such as anxiety and depression with mindfulness and EI.

### Mindfulness, EI, Anxiety and Depression: A Mediation Relationship?

In adults, there is a growing body of evidence that mindfulness exerts its beneficial effects on psychological wellbeing by influencing EI. Specifically, EI has been shown to mediate the relationship between: mindfulness and perceived stress (Charoensukmongkol, 2014; Bao et al., 2015); mindfulness and general self-efficacy (Charoensukmongkol, 2014); mindfulness and life satisfaction and mental distress (Wang and Kong, 2013); and mindfulness and subjective well-being (Schutte and Malouff, 2011).

The proposed theoretical explanation of this is that mindfulness increases self-awareness which leads to increased emotional self-control (Vago and Silbersweig, 2012), which then leads to greater well-being and less anxiety and depression. Research suggests that successful emotional regulation prevents both avoidance or over-engagement with emotions which are two tendencies that are associated with poor mental health outcomes (Beevers et al., 1999; Gross, 2002; Hu et al., 2014). The biological mechanism postulated to explain this effect is that mindfulness is associated with increased gray matter in brain areas associated with attention, learning, memory, and emotional regulation (Hölzel et al., 2011a,b; Paul et al., 2013; Marchand, 2014; Tang et al., 2015).

The current study extends this line of research in two important ways. First, this is the first study to investigate whether EI mediates the relationships between mindfulness, depression and anxiety. Secondly, this is the first study to investigate the relationships between EI, mindfulness, depression and anxiety in an adolescent population. This is important because rates of anxiety and depression are high in the adolescent population, and both conditions are associated with a range of negative outcomes. The identification of protective interventions is therefore of vital importance. The practice of mindfulness is one such putative intervention.

Recent studies have provided a more meaningful interpretation of EI’s influence on wellbeing variables by focusing on the subscale scores of EI, rather than the global EI scores. This study also focuses on subscale scores rather than global EI, to identify those aspects of EI which are most important in predicting depression and anxiety in adolescents.

## Materials and Methods

### Participants

One hundred and thirty-five Year 8 students from a public school in South East Melbourne were recruited to complete an online questionnaire. Of this sample, 108 participants were included in the analysis after 4 outliers and 23 cases with incomplete data were removed (*M*_age_ = 13.68, *SD*_age_ = 0.56, 51 males and 57 females). Incomplete data was mainly observed near the completion of the survey and was due to time constraints in terms of completing the survey in class-time.

### Measures

The online questionnaire consisted of five demographic questions and four scales measuring mindfulness, EI, anxiety and depression. These scales were selected as they have all been validated with an adolescent cohort and have previously been reported to show high validity and reliability.

### Dispositional Mindfulness

Dispositional mindfulness was measured using the Child and Adolescent Mindfulness Measure (CAMM; Greco et al., 2011). The CAMM is a 10-item self-report measure designed to assess three of the four facets of mindfulness assessed in adult mindfulness measures: *Observing*, which is the degree to which individuals attend to internal phenomena such as thoughts, feelings and bodily sensations (e.g., “I keep myself busy so I don’t notice my thoughts or feelings”); *Acting with awareness* which is present-centered awareness and full engagement in one’s current activity (e.g., “At school, I walk from class to class without noticing what I’m doing”); and *Accepting without judgment* which is non-judgmental awareness and openness to experiencing a full range of internal events (e.g., “I get upset with myself for having certain thoughts”). The fourth facet in adult mindfulness measures - *Describing*, which is the ability to put internal experiences into words - is not measured by the CAMM due to the probable impact of participant’s developmental level on their responses. The CAMM has been found to be a reliable and valid measure of dispositional mindfulness in a non-clinical sample of adolescents, with excellent convergent and incremental validity and with adequate internal consistency with Cronbach’s α = 0.81 (Greco et al., 2011). The CAMM correlates significantly and positively with outcomes such as quality of life and academic achievement, and negatively with internalizing symptoms and externalizing problem behavior (Greco et al., 2011).

### Adolescent EI

Adolescent EI was measured using the Adolescent Swinburne University Emotional Intelligence Test (Adolescent SUEIT; Luebbers et al., 2007). The Adolescent SUEIT is a 57-item test which measures four subscales of EI: Emotional Recognition and Expression (ERE; the ability to recognize one’s own emotions and express them to others – e.g., “I find it hard to talk about my feelings to other people”); Understanding Emotions (UE; the ability to identify and understand others’ emotions – e.g., “I can tell how others feel by the tone of their voice”); Emotions Direct Cognition (EDC; the use of emotions in decision-making and problem solving – e.g., “I use my feelings to help me find new ideas”); and Emotional Management and Control (EMC; the ability to manage and control emotions in oneself and in others – e.g., “I find it easy to control my anger and calm down”). Respondents indicated the extent to which each statement accorded with how they typically think, feel and act via a 5-point Likert scale ranging from 1 (very seldom) to 5 (very often). Negatively worded items were reverse scored and then scores were added together to give a total score for each scale. Subscale scores were added together to give a total EI score. Higher subscale scores indicate greater proficiency in that EI skill. A higher total score indicates greater EI. The Adolescent SUEIT has been found to be a reliable scale for total EI as well as for each subscale, with coefficient alpha’s ranging from α = 0.75 to α = 0.85 (Luebbers et al., 2007). The Adolescent SUEIT total EI score and subscales have found to be predictive of scholastic success (Downey et al., 2008b, 2013), bullying behavior and pro-victim attitudes (Schokman et al., 2014) and coping styles (Downey et al., 2010). In addition, the adult version of the A-SUEIT has been reported to show minimal social desirability (Downey et al., 2006).

### Anxiety

Anxiety was measured using the Beck Anxiety Inventory (BAI; Beck et al., 1988). The BAI is a 21-item self-report inventory used to assess anxiety levels in adults and adolescents. Respondents indicate to what extent they have been bothered by anxiety symptoms (e.g., “Numbness or tingling”) during the last month on a 4-point Likert scale ranging from 0 (Not at all) to 3 (Severely – it bothered me a lot). Scores on the 21-items are summed, and higher scores indicate higher anxiety. The BAI has been found to be a valid and reliable measure of anxiety, with an excellent internal consistency with Cronbach’s α = 0.92 (Beck et al., 1988). Construct validity studies show excellent convergence of the BAI with other measures of anxiety, including the STAI (*r* = 0.47–0.58) (Fydrich et al., 1992) and the Hamilton Anxiety Rating Scale (*r* = 0.56) (Beck and Steer, 1991).

### Depression

Depression was measured using the Beck Depression Inventory-II (BDI-II; Beck et al., 1996). The BDI-II is a 21-item self-report inventory used for detecting depression in adolescents and adults. The questionnaire consists of 21 depression symptoms (e.g., “self-dislike”), and respondents are required to pick one of four statements which best describes how they have been feeling in the past 2 weeks (e.g., 0 = “I feel the same about myself as ever” or 3 = “I dislike myself”). Two items were removed from the BDI for the purposes of the survey and analysis; a question relating to suicidal thoughts and wishes (question 9), and a question relating to loss of interest in sex (question 21). These questions were removed as the participant cohort was aged 13–15 years and the questions may have caused undue distress or discomfort. Scores were summed. High scores indicate more depression. Coefficient alpha estimates of reliability for the BDI-II with outpatients was 0.92 and was 0.93 for a non-clinical sample (Beck et al., 1996). Concurrent validity studies report that the BDI correlates with many other measures of depression including the SCL-90-D (Wang and Gorenstein, 2013) and the Revised Hamilton Rating Scale for Depression (Beck et al., 1996).

### Data Collection

This study was approved by the Swinburne University Human Research Ethics Committee (SHR Project 2016/112) and the Victorian Department of Education and Training (2016_003128).

### Statistical Analyses

**FIGURE 1:**
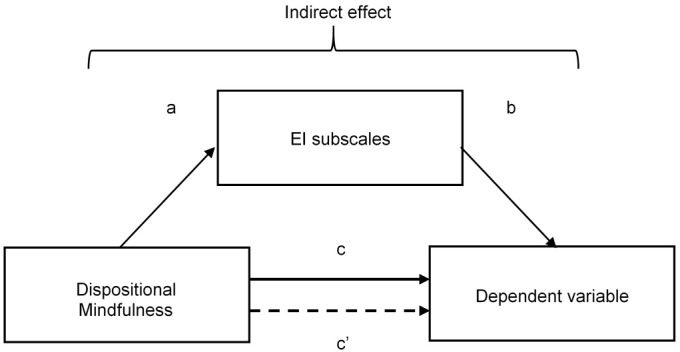
Mediation model with the predictor variable of dispositional mindfulness and the dependent variable of either anxiety or depression, mediated by El subscales. c, total effect in simple model where the mediator is not present; c’, direct effect in mediation model where the mediator is present.

We examined two multiple mediation models. The first the Sobel test is used in conjunction with the causal-steps method to test the significance of the mediation effect by computing the ratio of *ab* to its estimated standard error (Sobel, 1986). However the *p* value for this ratio is computed in reference to the standard normal distribution, and the sampling distribution of *ab* is only normal in large samples (Hayes, 2009). Preacher and Hayes (2008) advocate a mediation analysis technique which uses bootstrapping methods to compute confidence intervals for the indirect effect. The bootstrapping method is a non-parametric test which involves estimating a statistic by repeatedly randomly sampling observations with replacement from the data. As such, this method does not rely upon the assumption of normality and is therefore recommended for smaller sample sizes or skewed data. If zero does not fall between the resulting lower- and upper-bound confidence intervals of the bootstrapping method, the researcher can conclude that the indirect effect is not zero with CI% confidence. Perfect mediation is said to occur when *c’* is zero, which means that the relationship between the predictor and outcome is completely nulled by including the mediator in the model. Full mediation is unusual in social science research (Field, 2013), so we hypothesize that EI will partially mediate the relationships between mindfulness and anxiety and between mindfulness and depression. Two different indicators are used to measure the size of the indirect effect. The first uses the ratio of the indirect to the total effect (*P*_M_) which is calculated by multiplying the ‘a’ and ‘b’ paths from Figure 1 (i.e., the indirect effect) and dividing by the ‘c’ path (i.e., the total effect) (Preacher and Kelley, 2011). However caution should be taken when interpreting this measure as it can be unstable in samples smaller than 500, and the current study uses a sample of 108 (MacKinnon, 2008). For this reason, the *index of mediation* will also be reported to allow for comparison between mediators. The index of mediation standardizes the indirect effect with respect to the predictor, mediator and outcome variable (Field, 2013), and is useful in that it can be compared across different mediation models that use different measures of the predictor, mediator and outcome. Similar to the unstandardized indirect effect, if the lower and upper bound confidence intervals do not contain zero, then it can be concluded that the true effect size is different from ‘no effect’ (Field, 2013). The model examined used anxiety as the dependent variable and the second model used depression as the dependent variable. Dispositional mindfulness was the predictor variable and EI subscales were included in both models as multiple mediator variables. Figure 1 is a diagram of the mediation models being tested.

## Results

### Descriptive Statistics

Descriptive statistics and Cronbach’s alpha coefficients of the Adolescent SUEIT, Adolescent SUEIT subscales, the CAMM scale, the BDI and BAI scales are presented in Table 1.

**Table 1:** Descriptive statistics and reliability statistics of variables.

Scale	*M*	*SD*	α
Adolescent SUEIT	176.20	14.56	0.75
Emotional recognition and expression	17.36	4.54	0.56
Understanding emotions	61.71	6.57	0.69
Emotions direct cognition	20.20	3.61	0.58
Emotional management and control	53.17	8.17	0.74
Child and adolescent mindfulness measure	20.96	6.82	0.80
Beck anxiety inventory	18.16	15.23	0.95
Beck depression inventory	13.31	11.76	0.96

*N = 108.*

The mean scores for the CAMM were slightly lower for the relative comparison group (grade 8 boys) in the standardization paper for this instrument (Greco et al., 2011). The mean scores for the Adolescent SUEIT global score appear to be slightly less than the mean reported by Luebbers et al. (2007), which may be due to the fact that the sample in that study consisted of nearly three times as many females to males compared to the current study, where 52.8% of the sample were female. Females typically have a greater overall EI than males (Mayer et al., 1999; Joseph and Newman, 2010; Smieja et al., 2014; Wojciechowski et al., 2014) possibly explaining the higher mean in Luebber’s research. The mean for the EMC score in the current research is substantially higher than the mean in Luebber’s research (*M* = 42.99), once again possibly explained by the higher number of males in the current sample compared to Luebber’s research. Males have scored slightly higher in EMC than females in previous research (Luebbers et al., 2007; Downey et al., 2010). The mean scores for the BDI were slightly greater in the current sample than for previous research (Osman et al., 2008) but fell within the same classification range (Low – mild mood disturbance), while the mean scores for the BAI were higher but still within the same classification band to previous research (low anxiety) (Fydrich et al., 1992; Osman et al., 1997; Osman et al., 2002). The slightly higher mean scores for the BDI and BAI in the current sample may be because anxiety and depression is increasing in the population, or because data collection mostly occurred during Term 4, a period typically filled with anxiety-provoking exams and assessments. The EI global, UE, EMC, CAMM, BAI and BDI scales demonstrated acceptable reliability, with Cronbach’s alphas above 0.7, although the alpha’s for both the BDI and BAI were very high, suggesting some redundancy in those scales. The internal consistency for the EDC and ERE subscales were unacceptably low, with Cronbach’s alpha of 0.58 and 0.56, respectively. Four items were therefore removed from the ERE subscale until Cronbach’s alpha reached an acceptable 0.76. Three items were removed from the EDC subscale until Cronbach’s alpha reached 0.62 which is acceptable for a scale with seven items (Field, 2013).

### Correlations

Pearson correlation coefficients were calculated between all variables. These are displayed in Table 2.

**Table 2:** Pearson correlations between variables.

		1	2	3	4	5	6	7	8
1	EI global	1							
2	ERE	0.70^**^	1						
3	UE	0.57^**^	0.08	1					
4	EDC	0.24^*^	0.02	0.03	1				
5	EMC	0.72^**^	0.48^**^	0.05	-0.22^*^	1			
6	Mindfulness	0.46^**^	0.47^**^	0.05	-0.08	0.51^**^	1		
7	Anxiety	-0.44^**^	-0.51^**^	-0.09	0.19^*^	-0.42^**^	-0.45^**^	1	
8	Depression	-0.59^**^	-0.59^**^	-0.06	-0.09	-0.58^**^	-0.58^**^	0.66^**^	1

*N = 108. ERE, emotional recognition and expression; UE, understanding emotion; EDC, emotion directs cognition; EMC, emotional management and control. ^∗^p < 0.05; ^∗∗^p < 0.01, two-tailed.*

Pearson’s correlations revealed a significant moderate negative correlation between dispositional mindfulness and anxiety (*r* = -0.45) with a medium to large effect size (Cohen, 1992). *R^2^* = 0.20, meaning that 20% of the variance of mindfulness is shared with anxiety.

Pearson’s correlations revealed a significant negative correlation between dispositional mindfulness and depression (*r* = -0.58) which is a large effect (Cohen, 1992). *R^2^* = 0.34, meaning that 34% of the variance between mindfulness and depression is shared. Pearson’s correlations revealed significant negative relations between ERE (*r* = -0.51), and EMC (*r* = -0.42) sharing 26% and 18% of the variance with anxiety, respectively, both with medium to large effects.

Pearson’s correlations revealed that depression was significantly and negatively associated with ERE (r = -0.59) meaning ERE shares 35% of the variance with depression. This was a large effect (Cohen, 1992). EMC was also significantly and negatively associated with depression (*r* = -0.58, a large effect). Pearson’s correlations revealed significant positive and large correlations between ERE (*r* = 0.47) and EMC (*r* = 0.51) and mindfulness, which share 22 and 26% of the variance with mindfulness, respectively. Both were large effects. Pearson’s correlations revealed a significant positive and large relation between anxiety and depression (*r* = 0.66). *R^2^* = 0.44, meaning that anxiety and depression share 44% variance.

### Regression Analyses

Regression analysis was used to test the hypothesis that higher mindfulness, ERE and EMC would predict lower anxiety in adolescents. It was found that altogether, mindfulness and the all four EI subscales (ERE, UE, EDC, and EMC) did significantly predict lower anxiety, *F*(5,102) = 10.65, *p* < 0.001. The model accounted for 34% of the variance in adolescent anxiety (*R*^2^ = 0.34). Mindfulness (*b* = -0.46, *p* = 0.04) and ERE (*b* = -1.14, *p* < 0.001) were both significant predictors in the model, with mindfulness uniquely accounting for 3% of the variability of anxiety (*sr^2^* = 0.03), and ERE accounting for 8% of the variability of anxiety (*sr^2^* = 0.08). EMC was not a significant predictor in the model. We also predicted that higher mindfulness, ERE and EMC would predict lower depression in adolescents. It was found that altogether, mindfulness and the four EI subscales (ERE, UE, EDC and EMC) did significantly predict lower depression, *F*(5,102) = 22.03, *p* < 0.001. The model accounted for 52% of the variance in adolescent depression (*R*^2^ = 0.52). Mindfulness (*b* = -0.50, *p* < 0.001), ERE (*b* = -0.84, *p* < 0.001), and EMC (*b* = -0.40, *p* = 0.002) were all significant predictors in the model uniquely accounting for 6%, 7% and 5% of the variance of depression, respectively. It should be noted that these analyses are based on cross-sectional data rather than multiple time points. Caution should be exercised in interpreting cross-sectional data such as these. Future studies should examine these relationships with longitudinal data.

### Multiple Mediation Analysis

**FIGURE 2:**
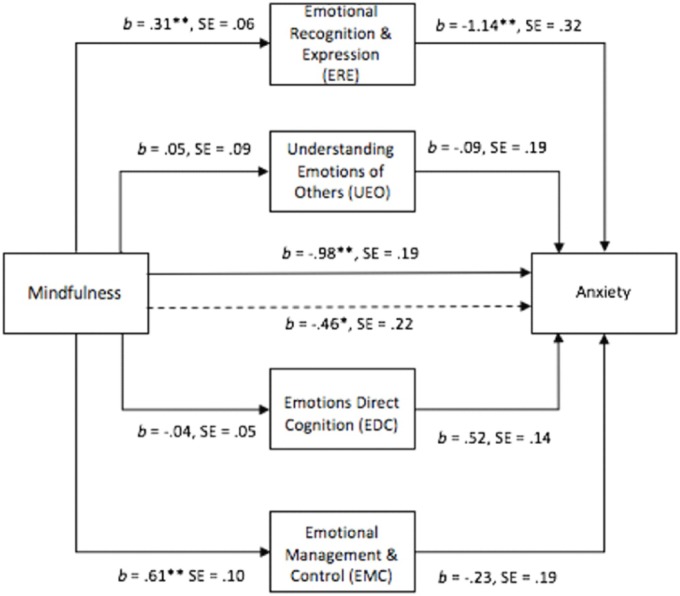
Multiple mediator model examining relation between dispositional mindfulness, subscales of El and anxiety. ^∗^*p* < 0.05; ^∗∗^*p* < 0.001.

Figure 2 displays the first multiple mediation model in which anxiety was the dependent variable and the EI subscales the mediator variables. Shown are the associated ‘a’ and ‘b’ paths (unstandardized coefficients), their standard errors, and the direct and total effects. A bootstrap sample of 1000 was specified. Figure 2 displays the results for the indirect effects and effect sizes for each pathway between mindfulness and anxiety, with EI subscales as the mediating variables.

**FIGURE 3:**
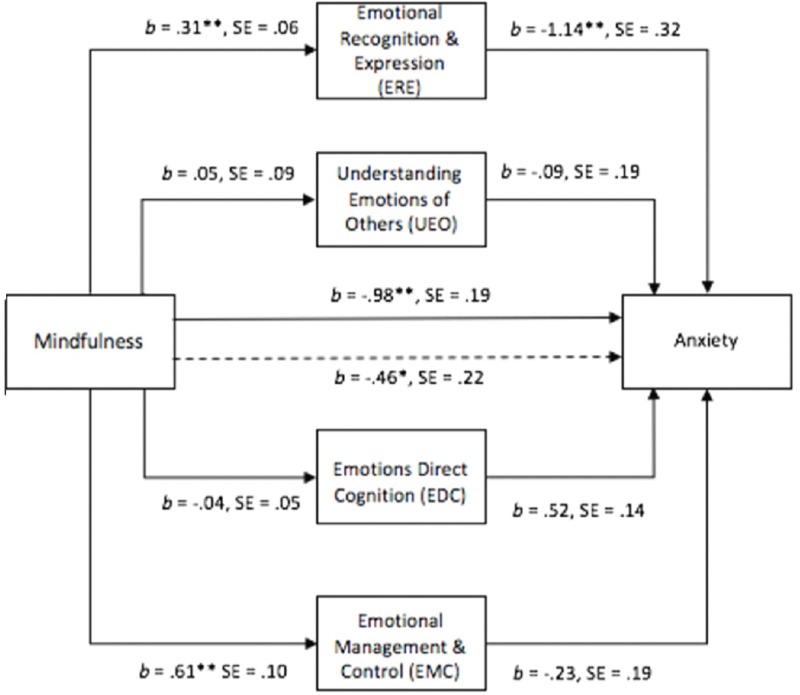
Multiple mediator model examining relation between dispositional mindfulness, subscales of Ei and anxiety. ^∗^*p* < 0.05; ^∗∗^*p* < 0.001.

Without any mediators in the model, the path between mindfulness and anxiety (path c in Figure 3) was significant. When all four EI subscales were included in the model, the path between mindfulness and anxiety (path c’) was also significant. Dispositional mindfulness had significant and positive direct paths to ERE and EMC but did not have significant direct paths to EDC or UE. Only ERE had a significant, negative direct path to anxiety. Therefore, the relation between mindfulness and anxiety was partially mediated by ERE. The direction of the paths suggests that mindfulness increases ERE and ERE decreases anxiety.

Figure 3 displays the second multiple mediation model where depression is the dependent variable and the EI subscales are the mediator variables. Shown are the associated ‘a’ and ‘b’ paths (unstandardized coefficients), their standard errors, and the direct and total effects. A bootstrap sample of 1000 was specified. Table 3 displays the results for the indirect effects effect sizes, and partial mediation for each pathway between mindfulness and depression, with EI subscales as the mediating variables.

**Table 3:** Indirect effect and sizes for model pathways between mindfulness and anxiety with EI subscales as the mediating variables.

Model pathway	Indirect effect	Index of mediation	PM
Mindfulness→ERE→Anxiety	-0.35, 95% CI [-0.65, -0.16]	-0.16, 95% CI [-0.30, -0.08]	0.36 95% CI [0.16, 0.73]
Mindfulness→UE→Anxiety	-0.004, 95% CI [-0.11, 0.02]	-0.002, 95% CI [-0.05, 0.01]	0.004, 95% CI [-0.03, 0.12]
Mindfulness→EDC→Anxiety	-0.02, 95% CI [-0.14, 0.05]	-0.01, 95% CI [-0.06, 0.02]	0.02, 95% CI [-0.05, 0.15]
Mindfulness→EMC→Anxiety	-0.14, 95% CI [-0.44, 0.06]	-0.06, 95% CI [-0.20, 0.03]	0.15, 95% CI [-0.07, 0.46]

In the depression model without mediators, the path between mindfulness and depression, (path c in Figure 3) was again significant. When all four mediators were included in the model, the path between mindfulness and anxiety (path c’) was also significant. The first part of this model is identical to the first model; that is, mindfulness had significant and positive direct paths to ERE and EMC but did not have significant direct paths to EDC or UE. ERE and EMC in this model had significant, negative direct paths to depression. This model suggests that mindfulness leads to increased ERE and EMC which leads to decreased depression (see Table 4).

**Table 4:** Indirect effect and sizes for model pathways between mindfulness and depression with EI subscales as the mediating variables.

Model pathway	Indirect effect	Index of mediation	PM
Mindfulness→ERE→Depression	-0.26, 95% CI [-0.49, -0.09]	-0.15, 95% CI [-0.28, -0.05]	0.26, 95% CI [0.08, 0.50]
Mindfulness→UE→Depression	-0.001, 95% CI [-0.03, 0.02]	-0.001, 95% CI [-0.02, 0.01]	0.001, 95% CI [-0.02, 0.04]
Mindfulness→EDC→Depression	0.001, 95% CI [-0.03, 0.05]	0.00, 95% CI [-0.02, 0.02]	-0.001, 95% CI [-0.05, 0.03]
Mindfulness→EMC→Depression	-0.24, 95% CI [-0.46, -0.10]	-0.14, 95% CI [-0.25, -0.05]	0.24, 95% CI [0.09, 0.42]

## Discussion

### Overview of Aims and Findings

Clinical and sub-clinical levels of anxiety and depression are prevalent in the adolescent population (Lawrence et al., 2015). Left untreated, these disorders can have serious negative long-term impacts on the individual and on society (Robins et al., 1996; Akse et al., 2004; van Aken and Semon Dubas, 2007; Goodman et al., 2011). For this reason, it is important to explore the correlates of good mental health, and whether there are interventions that can decrease anxiety and depression in adolescents.

Higher levels of dispositional mindfulness have been found to be associated with decreased depression (Hayes and Greco, 2008; Thompson and Gauntlett-Gilbert, 2008; Broderick and Blewitt, 2012) and decreased anxiety in adolescents (Semple et al., 2005; Bögels et al., 2008; Hayes and Greco, 2008; Thompson and Gauntlett-Gilbert, 2008). Importantly, dispositional mindfulness can be increased through practice via mindfulness meditation and other techniques (Carmody and Baer, 2008; Chu, 2010; Hassed, 2016).

Previous research has demonstrated the mediating effect of EI on the relation between mindfulness and various wellbeing variables: mindfulness and perceived stress (Charoensukmongkol, 2014; Bao et al., 2015); mindfulness and general self-efficacy (Charoensukmongkol, 2014); mindfulness and life satisfaction and mental distress (Wang and Kong, 2013); and mindfulness and subjective well-being (Schutte and Malouff, 2011). The current study extended this line of research to investigate whether specific EI subscales mediated the relation between mindfulness and anxiety, and mindfulness and depression in adolescents. The theoretical underpinning of these hypotheses is that the awareness and attention aspects of mindfulness may facilitate the development of greater EI by increasing gray matter in areas of the brain responsible for attention, focus and emotional processing, and this in turn decreases anxiety and depression.

In the current study as hypothesized, there was a strong positive association between anxiety and depression, meaning that adolescents with high anxiety were much more likely to also have high depression. Also, as expected, higher dispositional mindfulness was associated with lower anxiety and lower depression in adolescents. The associations between EI, mindfulness, anxiety and depression tell a more complex story however. While ERE and EMC both had the predicted strong negative associations with anxiety and depression and a positive association with mindfulness, EDC had a significant but weak positive association with anxiety. As predicted, UE was not associated with anxiety, depression, nor mindfulness. As hypothesized, both ERE and EMC partially mediated the relation between mindfulness and depression, but only ERE was found to partially mediate the relation between mindfulness and anxiety. EDC and UE mediated neither the relation between mindfulness and anxiety, nor the relation between mindfulness and depression as predicted.

### Implications and Future Research

The current study confirms several hypotheses – that mindfulness is associated with emotional intelligence, that depression and anxiety frequently co-occur in the adolescent population, and that the mechanism by which mindfulness impacts upon depression and anxiety is partially mediated by EI. These results are important because they demonstrate that increased mindfulness and increased EI are both associated with decreased anxiety and depression, but further experimental research must be conducted to confirm the causal hypothesis that mindfulness increases EI which then decreases anxiety and depression. Such research could involve a randomized control trial introducing a mindfulness intervention to a randomly selected group of adolescents versus a control group who undertake relaxation training. Baseline measurements of dispositional mindfulness, EI, anxiety and depression should be taken before introduction of the mindfulness intervention, during the intervention and a short period after the intervention has been completed.

Studies which investigate the duration and intensity of mindfulness training necessary to get positive results would also be useful, to ensure that mindfulness training is conducted in the most efficient manner possible. Longitudinal designs would also be useful to investigate changes in mindfulness, EI, anxiety, depression over time.

Another important implication of this research is that mindfulness training may improve EI which is important for many other outcomes, as EI has also been associated with more effective leadership (Batool, 2013; Siegling et al., 2014), higher resilience (Schneider et al., 2013) and better social, family, and intimate relationships (Mayer et al., 2008a).

As the subscales of EI only partially mediated the relations between mindfulness and anxiety and mindfulness and depression, it would be useful and interesting to investigate other mediators in the mindfulness – anxiety/depression relation using more sophisticated statistical techniques such as structural equation modeling. Additional mediators could include stress, attention, and relationship quality – all demonstrated to be associated with mindfulness in previous research (Baer et al., 2012; Bao et al., 2015; Nezlek et al., 2016).

## Conclusion

The current study investigated the relation between dispositional mindfulness, emotional intelligence, depression, and anxiety in an adolescent sample. The results of this study provide empirical support to previous research in adult samples suggesting that higher mindfulness is related to higher EI. Specifically, the two models in the current study suggest that greater mindfulness is related to a higher ability to recognize, express and manage emotions, and to lower levels of anxiety and depression. This is the first known study to examine the mediating effects of EI on the relations between mindfulness and anxiety and mindfulness and depression, and the first study to examine the relation between mindfulness, EI, anxiety and depression in an adolescent sample. Based on these results, future research should examine whether mindfulness could be manipulated to increase EI and reduce anxiety and depression in adolescents, thereby addressing a significant public health concern.

## Author Contributions

BF was involved in the conceptualization, data collection, data analysis, interpretation, and writing. JL was involved in the conceptualization, ethical approval, and data collection. LD was involved in the data analysis and writing. CS was involved in the conceptualization, ethical approval, data analysis, interpretation, and manuscript preparation.

## Conflict of Interest Statement

The authors declare that the research was conducted in the absence of any commercial or financial relationships that could be construed as a potential conflict of interest.
